# Oral Health Knowledge Gaps and Their Impact on the Role of Pediatricians: A Multicentric Study

**DOI:** 10.3390/ijerph181910237

**Published:** 2021-09-29

**Authors:** Murad Alrashdi, Maria-Eleni Limaki, Abdualelah Alrashidi

**Affiliations:** 1 Department of Orthodontic and Pediatric Dentistry, College of Dentistry, Qassim University, Buraydah 52571, Saudi Arabia; 2 251 Airforce General Hospital, Hellenic Air Force, 11525 Athens, Greece; limakicr@yahoo.com; 3 King Fahad Medical City, Riyadh 11525, Saudi Arabia; adalrashidi450@gmail.com

**Keywords:** oral knowledge, pediatrics, Saudi Arabia, Greece, United States

## Abstract

Purpose: This study aimed to evaluate the current level of pediatricians to promote oral health. In particular, the study sought to determine whether years of experience were associated with the dentistry knowledge of pediatricians. Materials and Methods: Online recruitment was used to obtain a sample of pediatricians from the United States of America, Greece, and Saudi Arabia. These three countries are the participants in this research project. The participants completed an anonymous, online, self-administered questionnaire. This questionnaire is available upon request. The differences in responses to knowledge questions, attitude questions, and solution questions were examined with respect to years of experience. Poisson regression models were used to examine whether these differences were statistically significant. Results: A total of 313 pediatricians participated in the study. The study found moderate levels of dental knowledge among pediatricians. A total of 53.4% reported that they had adequate knowledge to make the right recommendations on oral health for patients and parents. Compared to the participants in a residency program, the participants with 5 to 10 years of experience were over 2.72 times as likely to report adequate knowledge, and participants with 10 years of experience or more were nearly 1.98 times as likely to report adequate knowledge. There was a significant association between dentistry knowledge questions and attitude. Conclusion: The current level of influence of pediatricians in promoting pediatric oral health is limited to moderate. The gaps in oral health knowledge remain an issue, even among a broad sample of pediatricians from Greece, Saudi Arabia, and the United States, particularly pediatricians with less work experience.

## 1. Introduction

Protecting the dental health of children is an important part of children’s overall health and well-being [1,2]. It has been argued that oral health should be a more consistent part of pediatric medicine [3]. The failure to integrate oral health into pediatric practices can directly lead to poorer health outcomes. Dental caries in the primary dentition can have significant damaging effects on a child’s growth, due to the impairment of their oral functions [4]. Particularly, children from disadvantaged backgrounds are often most vulnerable to these dental health issues [5,6,7]. Since the first encounter of a child in a medical environment is often through pediatricians and medical practitioners, it is important that they are aware of the prevention of oral disease that begins early in life. Pediatricians are in a unique position to ensure that parents and other caregivers receive information on the prevention of oral disease in infants and young children. By working together, pediatricians and pediatric dentists can reinforce each other’s efforts to provide excellent preventive oral care [8]. Pediatricians, as the primary care providers for children, have the opportunity to make an important contribution to the overall health of their patients by improving their knowledge of oral disease and oral health and incorporating oral health into the daily routine of good childcare [9]. Unlike dentists, pediatricians see the majority of children periodically during their first years of life and have the opportunity to sensitize parents to the oral health of their children. Pediatricians play an important role in healthy growth and can provide effective guidance and preventive counseling about oral hygiene, diet, and fluoride exposure [10,11,12,13,14].

There is evidence that there may be knowledge gaps with respect to pediatric dentistry. A national survey demonstrated that many pediatricians lack the current scientific knowledge that is needed to promote children’s oral health. Only 39.5% knew about the transmissibility of dental caries; 37.3% understood that dental sealants are not usually applied to primary teeth; 60.8% knew the correct fluoride dosage for 4-month-old infants; and 22% were aware of fluoride varnishes. Only 9% answered four knowledge questions correctly [15]. Some pediatricians may not be identifying dental health issues and referring child patients. This prevents early intervention, which is an important element of dental care for children [12,13]. This may be due to some pediatricians attaching a low importance to dental health [14]. A previous study documented moderate levels of knowledge of oral health among pediatricians [16]. Additionally, dental care between pediatricians and dentists is often not well-coordinated. For example, one study found that many pediatricians did not believe that referral to a dentist was needed as soon as dental issues were identified [17]. A study that examined how oral health can be better integrated as part of pediatric care suggested that increasing the training in oral health among pediatric dentists may be an effective strategy to confront these deficiencies [18]. Therefore, pediatricians must be competent in the issues of oral health and disease if they are to fulfill their roles as professionals that are dedicated to the health of children.

In 2003, the American Academy of Pediatrics (AAP) crafted a policy statement entitled “Oral Health Risk Assessment Timing and Establishment of the Dental Home” [19]. While this policy has been in place since 2003, we hypothesize that pediatricians still face many barriers to comply with it. Pediatricians are one of the main points of health-related education for children and their parents. Therefore, pediatricians can play an important role with respect to dental health. This study sought to further understand the factors that impact the knowledge gaps among pediatricians. In order to understand these factor, this study examined how pediatricians’ knowledge differs according to their years of experience.

## 2. Materials and Methods

### 2.1. Study Design

This study used a cross-sectional multicenter design involving dental pediatricians from three different countries, including the United States of America, Greece, and Saudi Arabia. The inclusion criteria for this study was that the respondents needed to be pediatricians who were practicing in either the United States, Saudi Arabia, or Greece. Pediatricians from other countries and non-pediatric clinicians were excluded.

### 2.2. Study Period

The study period was from 1 January 2020to 30 June 2020, during which the participants completed the questionnaire in the selected three countries.

### 2.3. Instrument

An anonymous, online, self-administered questionnaire was developed by the researchers. This instrument can be provided upon request. The questionnaire, with an informed consent statement, was distributed among the pediatricians who were practicing in the three countries. The questionnaire variables included demographic data, attitude, knowledge, and perspectives about proposed solutions toward pediatric oral health. The questionnaire was adapted from the AAP Periodic Survey of Fellows #70, which focuses on oral health within pediatric medical clinics [20]. Questionnaire items were developed by the AAP Division of Health Services Research and experts from the AAP team to improve oral health disparities in early childhood. The survey addressed pediatricians’ practices and the barriers to dental health assessment, consultation, and referrals of young patients from birth to 3 years of age.

### 2.4. Sampling

Participants were identified for participation by random email. These emails were sent by a pediatrician at the University of California in Los Angeles, USA, a pediatric resident at King Fahad Medical City in Saudi Arabia, and a pediatrician at University of Athens Medical school. The pediatricians were identified using a convivence sample approach. A total of 452 (161 in USA, 141 in Saudi Arabia, 150 in Greece) pediatricians were sent emails. The sample was a convenience sample. It was not designed to be representative of the relevant populations, but rather to generate knowledge of the situation in these countries.

### 2.5. Statistical Analysis

Differences in attitude, knowledge, and perspectives about the proposed solutions toward pediatric oral health (dependent variables) were examined based on country, gender, and patient age (independent variables). Whether these differences were significant was assessed using Chi-square analysis. In a similar way, the probability of agreeing with dentistry attitude, knowledge, and solution questions, according to experience, was examined using Poisson regression. The Relative Risk (RR) in these models represents how many times higher the probability of agreeing with a particular statement is among each group, compared to the reference group. For this analysis, a univariate model that only included experience, and an additional multivariable model that controlled for the country, gender, and patient age, were constructed. Finally, answers to knowledge questions were associated with attitudes and proposed solutions. Differences in responses were tested using Chi-square analysis. All analyses were performed using SAS Version 9.3 (SAS Institute Inc., Cary, NC, USA).

## 3. Results

### 3.1. Demographic Distribution of the Participants

A total of 313 participants were included in the study, with respondents from the United States (106), Saudi Arabia (108), and Greece (99), and a response rate of 69%. The respondents’ results were included in this survey if they met the inclusion criteria of being practicing pediatricians in one of the three countries that were surveyed. This was a convenience sample that was sent through email to 452 pediatricians. There are a total of 27,550 pediatricians in the United States [21], 2466 pediatricians in Saudi Arabia [22], and 4278 pediatricians in Greece [23]. There was also a near similar number of male and female respondents (male: 163, female: 150). Most of the respondents treated patients that were 1 month to 5 years old (196). Most of the respondents had 5 to 10 years of experience (92), followed by more than 10 years of experience (81). There was a fairly even distribution of the experience of the respondents, with the fewest number of respondents having 1 to 5 years of experience (65) and the most having 5 to 10 years of experience (92), as shown in Table 1.

### 3.2. Results of the Main Questionnaire

Table 2 shows the differences in attitude, knowledge, and solution questions according to years of experience. Respondents with 5 to 10 years of experience were significantly more likely to agree that, when they treat a child with a complicated medical history, they consult a pediatric dentist, due to concerns about the oral health of the child (RR = 1.80, 95% CI = 1.20, 2.72). This relationship was not significant in model 2, which controlled for experience, country, gender, and patient. The findings, not being significant in model 2, suggest that the relationship between experience and making this recommendation may be confounded by the other variables that were controlled in the model. The respondents with 5 to 10 years of experience were significantly more likely to have attended a class on oral health during their residency (RR = 3.54, 95% CI = 1.71, 7.29). This relationship remained significant for those with 5 to 10 years of experience in model 2 after controlling for country, gender, and patient age, which means that it is likely that experience has an effect on the probability of having attended these programs, and that the relationship was not confounded by other factors.

Respondents with 5 to 10 years of experience (RR = 2.72, 95% CI = 1.67, 4.43) and more than 10 years of experience (RR = 1.98, 95% CI = 1.18, 3.33) were significantly more likely to report having the adequate knowledge to make the right recommendations on oral health for their patients and their parents, compared to those in a residency program. This relationship remained significant in model 2. Those with 5 to 10 years of experience were significantly more likely to recommend wiping or brushing after breastfeeding or bottle feeding with milk (RR = 1.47, 95% CI = 1.02, 2.11). This relationship was not significant in model 2 (Table 2).

## 4. Discussion

This study addresses the importance of pediatricians’ role in the oral health of children. Using an international pool of pediatricians, the study identified that experience was generally associated with more knowledge with respect to the oral health of children. With respect to the specific objectives of the study, it was found that the pediatricians who were residents, or had fewer than 5 years of experience, tended to have lower knowledge. This finding suggests that education around pediatric dentistry, particularly in medical school, may be needed to improve knowledge among these cohorts. In a similar way, more knowledge was generally associated with better attitudes and a better perspective with respect to pediatric oral health.

These findings build upon previous research. Previous research has found that there may be deficiencies in knowledge with respect to pediatric dentistry among pediatricians [24]. Similar to previous research, this study also documented moderate levels of dental knowledge among pediatricians. This study indicates that there is variability in this knowledge and that different factors, particularly experience, are important contributors to this variability. Previous work has also examined dental referral practices among pediatric dentists [14,24,25,26]. This study also found that, in many cases, pediatricians are not referring their patients. However, pediatricians with more knowledge are more likely to make these referrals.

Similar to the findings from this study, a previous study that sought to assess the levels of knowledge among pediatricians with respect to dental health also found some areas in which knowledge was lacking [15,24]. For example, while most pediatricians answered the knowledge questions correctly, there was evidence that there was not sufficient knowledge among these pediatricians with respect to mother-to-child transmission of cavity-causing bacteria and dental sealants [15]. Additionally, the majority of pediatricians did not indicate that they were likely to apply fluoride, inquire about a mother’s dental health, or refer their patients to a dentist [24].

Another survey conducted in Canada found similar deficiencies in the knowledge of dental health among pediatricians [24]. In the survey, fewer than 1% of pediatricians answered all of the six knowledge questions correctly [24]. Similar to the findings from this study, only approximately 1/5th of pediatricians reported that they had received training in dental health while in medical school.

It was observed that pediatricians with more than 5 years of experience were more likely to recommend wiping or brushing after breastfeeding or bottle feeding with milk, compared to those with less experience (Table 2). The American Dental Association recommends this type of wiping following breastfeeding in order to protect the dental health of infants, particularly those who are growing their first set of teeth [25]. The previous research about pediatricians’ knowledge with respect to bottle/breastfeeding and dental health has shown some lack of knowledge among pediatricians. One study, in particular, found that the guidelines for bottle feeding were not always followed or comprehended by both pediatricians and pediatric dentists [26].

Different areas for intervention can be identified from this study. For example, among those with the highest knowledge score, nearly 70% reported that they had attended a class on oral health during residency. Including oral health classes and/or seminars during medical school, particularly for those who are interested in pediatrics, may be an effective way to improve the knowledge of oral health among pediatricians. In addition, incorporating oral health components into the well-child visit for pediatric medical care might be a good intervention to reduce this gap.

This study has some limitations. Since the participants were identified for participation by random emails sent from pediatricians, it is not possible to be certain that the findings are, in fact, representative of the pediatricians in each country, which may affect the generalizability of the findings. Therefore, caution should be made when assuming these results are generalizable to larger populations. In the future, using a probability sampling method may produce a representative sample. Additionally, the findings of this study are based on self-reports and may be vulnerable to self-report bias. Some respondents may provide certain answers that they perceive to be more socially acceptable. However, because the survey is anonymous, the effect of this potential bias is limited.

Efforts to build on these findings can include collecting data from a systematic sample, the use of other data collection methods (such as focus groups) to develop an understanding of how the knowledge of pediatric dentistry can be improved, and longitudinal methods. Longitudinal studies can be carried out to examine the predictors of better knowledge. Training in medical school is an important first step to improve this knowledge. Additionally, the healthcare system should seek to better integrate medical and dental practices.

## 5. Conclusions

Although these findings should be interpreted with caution, given the fact that they came from a convenience sample, they indicate that pediatricians have limited to moderate knowledge and understanding of pediatric oral health. However, pediatricians with more clinical experience were more knowledgeable than those with less experience. The barriers for pediatricians to promote oral health include inadequate education and training during medical school or residency, in addition to the lack of patient referral.

## Figures and Tables

**Table 1:** Experience in dentistry according to country, gender, and patient age.

	In a Residency Program	1 to 5 Years	5 to 10 Years	More Than 10 Years	Total	*p*-Value ^1^
	*n* (%)	*n* (%)	*n* (%)	*n* (%)		
**Country**						<0.0001
Greece	14 (14.1)	31 (31.3)	38 (38.4)	16 (16.2)	99	
Saudi Arabia	43 (40.6)	12 (11.3)	22 (20.1)	29 (27.4)	106	
USA	18 (16.7)	22 (20.4)	32 (29.6)	36 (33.3)	108	
**Gender**						0.2690
Female	34 (22.7)	28 (18.7)	52 (34.7)	36 (24.0)	150	
Male	41 (21.2)	37 (22.7)	40 (24.5)	45 (27.6)	163	
**Patient age**						0.0033
1 month to 5 years	42 (21.4)	49 (25.0)	60 (30.6)	45 (23.0)	196	
5 to 10 years	17 (30.9)	11 (20.0)	18 (32.7)	9 (16.4)	55	
10 to 15 years	16 (25.8)	5 (8.1)	14 (22.6)	27 (43.6)	62	
**Total**	**75 (24.0)**	**65 (20.8)**	**92 (29.4)**	**81 (25.9)**	**313**	

^1^—Derived from Chi-square analysis.

**Table 2:** Dentistry attitude, knowledge, and solution questions according to experience with regression models.

	Agree or Strongly Agree	Model 1		Model 2	
Oral Health is an essential part of overall health. *	N (%)	RR (95% CI)	*p*-value	RR (95% CI)	*p*-value
In a residency program	75 (100.0)				
1 to 5 years	65 (100.0)				
5 to 10 years	92 (100.0)				
More than 10 years	81 (100.0)				
**It is important to consult a pediatric dentist in my pediatric practice in terms of oral issues.**					
In a residency program	61 (81.3)	1 (reference)		1 (reference)	
1 to 5 years	54 (83.1)	1.02 (0.71, 1.47)	0.9096	0.88 (0.60, 1.28)	0.4953
5 to 10 years	82 (89.1)	1.10 (0.79, 1.53)	0.5882	0.94 (0.67, 1.32)	0.708
More than 10 years	65 (80.3)	0.99 (0.70, 1.40)	0.9399	0.91 (0.63, 1.30)	0.5867
**As a pediatrician, when I treat a child with a complicated medical history (liver disease, heart disease, special syndrome, etc.) I consult a pediatric dentist due to my concern about the oral health of the child.**					
In a residency program	33 (44.0)	1 (reference)		1 (reference)	
1 to 5 years	40 (61.5)	1.40 (0.88, 2.22)	0.1537	1.18 (0.73, 1.91)	0.4893
5 to 10 years	73 (79.4)	1.80 (1.20, 2.72)	0.0049	1.45 (0.95, 2.22)	0.0836
More than 10 years	37 (45.7)	1.04 (0.65, 1.66)	0.8757	0.86 (0.53, 1.39)	0.5347
**I have attended a class on oral health during residency.**					
In a residency program	9 (12.0)	1 (reference)		1 (reference)	
1 to 5 years	1 (1.5)	0.13 (0.02, 1.01)	0.0513	0.13 (0.02, 1.01)	0.0514
5 to 10 years	39 (42.4)	3.54 (1.71, 7.29)	0.0006	3.41 (1.61, 7.21)	0.0013
More than 10 years	20 (24.7)	2.06 (0.94, 4.52)	0.0722	1.46 (0.65, 3.27)	0.3531
**I have the adequate knowledge to make the right recommendations on oral health for my patients and their parents.**					
In a residency program	21 (28.0)	1 (reference)		1 (reference)	
1 to 5 years	31 (47.7)	1.70 (0.98, 2.96)	0.0595	1.45 (0.81, 2.57)	0.2075
5 to 10 years	70 (76.1)	2.72 (1.67, 4.43)	<0.0001	2.33 (1.41, 3.87)	0.001
More than 10 years	45 (55.6)	1.98 (1.18, 3.33)	0.0095	1.71 (1.01, 2.91)	0.0459
**I recommend wiping or brushing after breastfeeding or bottle feeding with milk, particularly at nighttime, as the first primary tooth begins to erupt.**					
In a residency program	45 (60.0)	1 (reference)		1 (reference)	
1 to 5 years	43 (66.2)	1.10 (0.73, 1.67)	0.6471	0.79 (0.51, 1.22)	0.2822
5 to 10 years	81 (88.0)	1.47 (1.02, 2.11)	0.0392	1.07 (0.74, 1.56)	0.709
More than 10 years	63 (77.8)	1.30 (0.88, 1.90)	0.1837	1.08 (0.73, 1.60)	0.6961
**I recommend the consumption of healthy snacks between meals and reduction of the sugar-containing snacks.**					
In a residency program	64 (85.3)	1 (reference)		1 (reference)	
1 to 5 years	53 (81.5)	0.96 (0.66, 1.38)	0.8065	0.83 (0.57, 1.22)	0.348
5 to 10 years	83 (90.2)	1.06 (0.76, 1.46)	0.7379	0.94 (0.67, 1.32)	0.7256
More than 10 years	74 (91.4)	1.07 (0.77, 1.50)	0.6894	1.00 (0.71, 1.41)	0.9985
**I recommend the correct amount of fluoridated toothpaste to be used twice daily for the prevention and control of caries.**					
In a residency program	58 (77.3)	1 (reference)		1 (reference)	
1 to 5 years	44 (67.7)	0.88 (0.59, 1.30)	0.5054	0.88 (0.58, 1.32)	0.5309
5 to 10 years	54 (58.7)	0.76 (0.52, 1.10)	0.1448	0.76 (0.51, 1.11)	0.1524
More than 10 years	80 (86.4)	1.12 (0.79, 1.58)	0.5315	1.05 (0.73, 1.51)	0.7924
**I recommend a dental visit should be established for all infants by 12 months of age.**					
In a residency program	54 (72.0)	1 (reference)		1 (reference)	
1 to 5 years	43 (66.2)	0.92 (0.62, 1.37)	0.6786	0.85 (0.56, 1.29)	0.4347
5 to 10 years	62 (67.4)	0.94 (0.65, 1.35)	0.7223	0.87 (0.60, 1.26)	0.4593
More than 10 years	68 (84.0)	1.17 (0.82, 1.67)	0.3995	1.12 (0.77, 1.62)	0.5532
**I recommend a rotation in pediatric dentistry during pediatric medical residency program.**					
In a residency program	45 (60.0)	1 (reference)		1 (reference)	
1 to 5 years	34 (52.3)	0.87 (0.56, 1.36)	0.546	0.64 (0.40, 1.02)	0.0599
5 to 10 years	63 (68.5)	1.14 (0.78, 1.67)	0.4983	0.86 (0.58, 1.28)	0.4572
More than 10 years	54 (66.7)	1.11 (0.75, 1.65)	0.6017	0.99 (0.66, 1.48)	0.9563
**Communication should be established between pediatricians and pediatric dentists to write a protocol/guideline for both parts in terms of pediatric oral health education.**					
In a residency program	67 (89.3)	1 (reference)		1 (reference)	
1 to 5 years	54 (83.1)	0.93 (0.65, 1.33)	0.6913	0.83 (0.57, 1.20)	0.3211
5 to 10 years	92 (100.0)	1.12 (0.82, 1.53)	0.4825	1.02 (0.73, 1.41)	0.927
More than 10 years	74 (91.4)	1.02 (0.73, 1.42)	0.8943	0.95 (0.68, 1.33)	0.7656
**Courses and continuing educational hours of pediatric dentistry should be mandatory for medical pediatricians during their career.**					
In a residency program	43 (57.3)	1 (reference)		1 (reference)	
1 to 5 years	43 (66.2)	1.15 (0.76, 1.76)	0.507	0.91 (0.58, 1.41)	0.6666
5 to 10 years	70 (76.1)	1.33 (0.91, 1.94)	0.1441	1.09 (0.73, 1.62)	0.6681
More than 10 years	48 (59.3)	1.03 (0.68, 1.56)	0.875	0.99 (0.65, 1.51)	0.9736
**I encourage the professional collaboration practice among pediatricians and pediatric dentists in health centers.**					
In a residency program	72 (96.0)	1 (reference)		1 (reference)	
1 to 5 years	54 (83.1)	0.87 (0.61, 1.23)	0.5067	0.80 (0.56, 1.16)	0.2485
5 to 10 years	92 (100.0)	1.04 (0.77, 1.42)	0.0466	0.97 (0.71, 1.34)	0.8756
More than 10 years	79 (97.5)	1.02 (0.74, 1.40)	0.2522	0.97 (0.70, 1.35)	0.8638

Model 1: Univariable. Model 2: Controlling for experience, country, gender, and patient age simultaneously. RR = Rate Ratio. *—RR cannot be calculated due to all respondents agreeing with statement.
